# An Analysis of Deep Learning Models in SSVEP-Based BCI: A Survey

**DOI:** 10.3390/brainsci13030483

**Published:** 2023-03-13

**Authors:** Dongcen Xu, Fengzhen Tang, Yiping Li, Qifeng Zhang, Xisheng Feng

**Affiliations:** 1 State Key Laboratory of Robotics, Shenyang Institute of Automation, Chinese Academy of Sciences, Shenyang 110016, China; xudongcen@sia.cn (D.X.); tangfengzhen@sia.cn (F.T.); lyp@sia.cn (Y.L.); zqf@sia.cn (Q.Z.); 2 Institutes for Robotics and Intelligent Manufacturing, Chinese Academy of Sciences, Shenyang 110169, China; 3 University of Chinese Academy of Sciences, Beijing 100049, China

**Keywords:** brain–computer interface, steady-state visual evoked potential, deep learning, convolutional neural networks

## Abstract

The brain–computer interface (BCI), which provides a new way for humans to directly communicate with robots without the involvement of the peripheral nervous system, has recently attracted much attention. Among all the BCI paradigms, BCIs based on steady-state visual evoked potentials (SSVEPs) have the highest information transfer rate (ITR) and the shortest training time. Meanwhile, deep learning has provided an effective and feasible solution for solving complex classification problems in many fields, and many researchers have started to apply deep learning to classify SSVEP signals. However, the designs of deep learning models vary drastically. There are many hyper-parameters that influence the performance of the model in an unpredictable way. This study surveyed 31 deep learning models (2011–2023) that were used to classify SSVEP signals and analyzed their design aspects including model input, model structure, performance measure, etc. Most of the studies that were surveyed in this paper were published in 2021 and 2022. This survey is an up-to-date design guide for researchers who are interested in using deep learning models to classify SSVEP signals.

## 1. Introduction

The brain–computer interface (BCI) provides a direct communication channel between the human brain and computers without using peripheral nerves or muscles [1]. BCIs allow users to harness their brain states for controlling devices such as spelling interfaces [2,3], wheelchairs [4,5], computer games [6,7], or other assistive devices [8,9]. Among all BCIs, electroencephalography (EEG)-based BCIs are the most widely used. EEG is a non-invasive way of acquiring brain signals from the surface of the human scalp and is widely adopted in brain–computer interface applications because of its safety, convenience, and high temporal resolution [10,11,12]. There are multiple commonly used paradigms to evoke brain signals to generate the control commands for EEG-based BCIs, including P300 [13], motor imagery [14], and steady-state visual evoked potential (SSVEP) [15]. 

Among them, SSVEP has the advantages of less training, high classification accuracy, and a high information transfer rate (ITR) [16] and is considered to be the most suitable paradigm for effective high-throughput BCI [17]. SSVEP represents oscillatory electrical potential that is elicited in the brain when the subject is visually watching a stimulus that is flickering at a frequency of 6 Hz or above. A reorganization of spontaneous intrinsic brain oscillations in response to a stimulus will likely take place [18]. These SSVEP signals are most evident in the occipital region (visual cortex) with the fundamental frequency being the same as the stimulus and its harmonics [19]. 

SSVEP-based BCIs generally consist of five main processing stages: the data collection stage that records neural data; the signal preprocessing stage that preprocesses and cleans the recorded data; the feature extraction stage that extracts meaningful information from the neural data; the classification stage that determines the output of the BCI from the processed neural data mostly using machine learning methods; and the feedback stage that presents the output of the BCI to the user [20]. 

Compared with other classification methods based on SSVEP, deep learning has many advantages. It integrates feature extraction and classification as a single process; therefore, deep learning is more likely to acquire subtle patterns that are not observable by humans but are informative for the classification of EEG signals. Deep learning utilizes a neural network consisting of several stacked layers of neurons, with each layer trained on a distinct set of features depending on the output of previous layers. As the data flow through the network, more complex features are obtained. The network can take raw SSVEP signals as the input, without the requirement for hand-crafted feature extraction as well as common signal preprocessing steps [21,22]. This property provides a critical advantage, as it precludes implicit EEG signals or features from being lost during preprocessing or feature extraction [23].

However, the design of deep learning models varies significantly, and it is hard to predict the performance of the model by its structure. The preprocessing of data, the number of neurons, the number of layers, the choice of activation functions, the choice of training methods, and the adoption of pooling layers or the dropout technique to prevent overfitting all impact the performance of the model, and thus surveying successful deep learning models and learning from their structures is of great significance for the designing of future deep learning models.

### 1.1. Related Surveys

The reviews and surveys on using deep learning models to classify SSVEP from 2019 to 2023 are summarized in Table 1. As shown in Table 1, most of the surveys did not cover the detailed deep structures or hyperparameters of the deep learning models, which are critical references for designing future deep learning models. Only Craik’s work covered these two areas in 2019; however, with the fast advancement of deep learning techniques, it is necessary to gather recent research results to offer up-to-date information for current researchers. This survey provides detailed deep learning model analysis which includes details of structures and hyperparameters for 31 deep learning models, most of which were published in 2021 and 2022. This survey is an up-to-date survey aimed at providing design details and design analysis for future deep learning models in SSVEP classification.

### 1.2. Literature Search and Inclusion Criteria

To conduct this survey, the following databases were used: PubMed, Engineering Village, ScienceDirect, IEEE Xplore, and Google Scholar. Papers were selected for survey if the following keywords appeared in their title: (1) SSVEP and (2) deep learning or an RNN or CNN or DNN or LSTM. 

After further reading, papers that satisfied the following criteria remained in this survey: (1) written in English; (2) had innovations in the structural design of deep learning models; (3) had detailed information regarding model input, structure, and performance (or at least 70% of the details revealed); and (4) the deep learning model was designed to classify SSVEP signals. After selection, 31 articles remained in this survey, and the 31 deep learning models were dissected and analyzed in detail in the following content.

### 1.3. Quality Assessment

**Figure 1:**
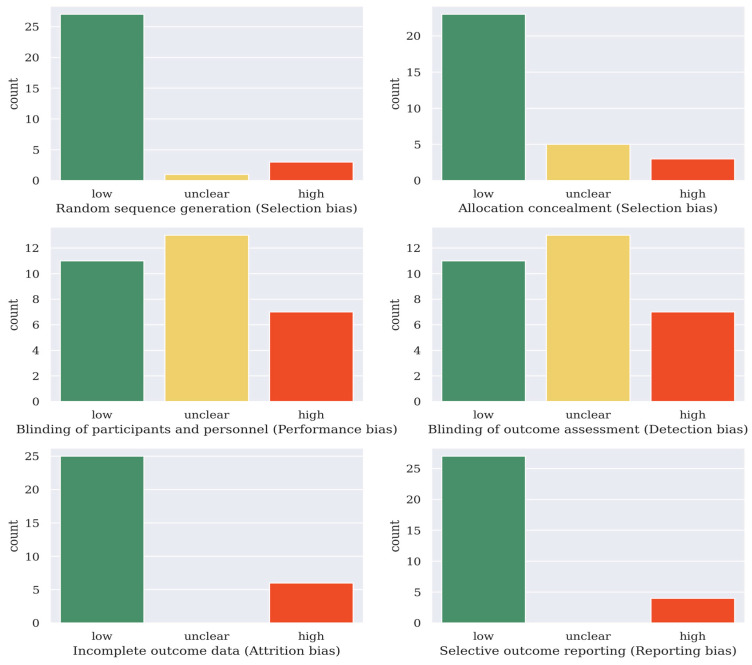
Risk of bias assessment result of the 31 deep learning articles covered in this survey (Cochrane’s collaboration tool [34]).

The Cochrane collaboration tool was used to assess the quality of the selected articles [34]. For the 31 articles included in this survey, they were classified into having (a) a low risk of bias, (b) a high risk of bias, or (c) an unclear risk of bias in six domains. The quality of the articles was categorized into weak (fewer than three low-risk domains), fair (three to five low-risk domains), or good (six low-risk domains). Of the 31 articles included in this survey, 4 of them were categorized as weak, 19 were categorized as fair, and 8 were categorized as good. The results are shown in Figure 1. 

### 1.4. Contribution of This Survey

Other than hand-crafted approaches such as feature extraction methods or machine learning methods, where mathematical calculation can help in predicting the performance of models, the performance of deep learning models is rather unpredictable, and the design process often includes a trial-and-error approach to validate the design choice of structure or hyperparameters. Thus, when using deep learning models as the classification method for SSVEP classification, the overview of detailed structures and hyperparameters of other successful deep learning models can significantly facilitate the design process, which is one of this survey’s key advantages.

To the best of our knowledge, this survey is the first one aimed at dissecting the deep learning models used for SSVEP signal classification in different aspects and providing a thorough design guide for future deep learning models targeting the classification of SSVEP signals since 2019. To this end, 31 deep learning models for SSVEP classification are dissected and analyzed in detail, most of which were published in 2021 and 2022. Three key contributions are made in this survey:

- Key elements of deep learning models are introduced to help readers gain a comprehensive understanding of deep learning models;
- Design details of 31 deep learning models are listed to provide information and handy references for the design of future deep learning models;
- Design considerations of deep learning models are analyzed and discussed, which can benefit: (1) researchers with a computer background who are interested in SSVEP-based BCI; (2) neuroscience experts who intend to construct deep learning models to classify SSVEP signals.

In sum, this survey provides a thorough and convenient guide for the future design of deep learning models for SSVEP classification.

### 1.5. Organization of This Survey

The rest of this survey is structured as follows: Section 2 introduces the model input and three frequently used open datasets of SSVEP signals, as well as the data preprocessing methods; Section 3 overviews model structure designs, including DNN models, long short-term memory (LSTM) models, CNN models and their components such as pooling layers, dropout, training methods, and activation functions; Section 4 discusses the design considerations and performance measures of models; Section 5 points out the current challenges and future directions; and Section 6 provides the concluding remarks.

## 2. Model Input

The quantity of training data has a crucial impact on the performance of deep learning models. The more complex a deep learning model, the more data it requires in training, otherwise its performance will not surpass a simpler deep learning model or traditional machine learning approaches [21]. In BCI research, the quantity of data can be measured by the SSVEP signal length per channel. The data lengths of the 31 deep learning studies are analyzed as references for researchers who want to collect their own data, and three frequently used public datasets are presented for researchers who are not capable of collecting SSVEP data. The preprocessing methods of input data are also introduced, as preprocessing of input data can make the features of data easier to extract, thus increasing the models’ performances. 

### 2.1. Data Length 

**Figure 2:**
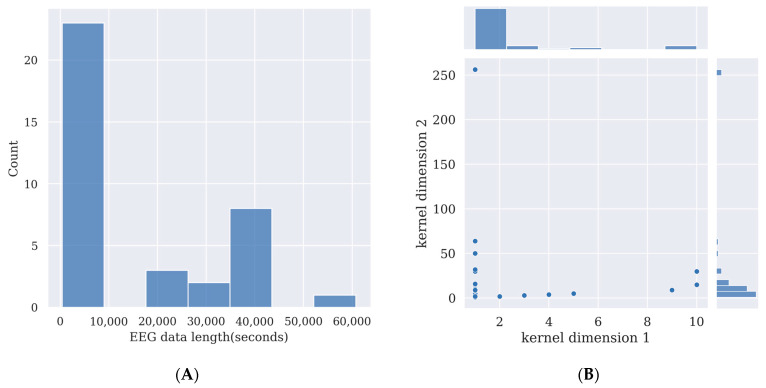
(**A**) A summary of steady-state visual evoked potential (SSVEP) data length per channel used by 31 deep learning studies, as shown in Table 2. (**B**) A summary of the kernel dimensions of convolutional layers in 31 deep learning studies, as shown in Table 2.

Deep learning models require the training of the models’ parameters. This usually requires a large amount of data. By using more data, the performance of the model will be enhanced. Additionally, the more complex a deep learning model is, and the more parameters there are in the model, the more data it requires to train it, otherwise its performance will not surpass simpler deep learning models or other feature extraction methods such as canonical correlation analysis (CCA), or machine learning methods such as support vector machine (SVM) [21]. However, recording EEG data from participants takes effort; thus, the size of the experimental dataset is limited. Here, the time length of the SSVEP signal in each channel in 31 deep learning studies is overviewed in Figure 2A to provide a guide for SSVEP signal length for researchers who want to prepare their own data for training deep learning models.

The more complex a deep learning model is, the more data it needs for training. If insufficient data are used for training the deep learning model, the model will learn slight variations and noise in the training data, which are exclusive to that database and do not reflect the features of the target signal. This is known as overfitting and will harm the model’s performance in testing while using data other than the training dataset [21]. As Figure 2A shows, for comparatively complex deep learning models, an SSVEP signal length between 40,000 s and 50,000 s may provide enough data to train the model if researchers want to collect their own data and use a model of a similar size to those covered in this survey. For relatively simple deep learning models, an SSVEP signal length below 10,000 s may be enough for training. Detailed data length and data point calculations are given in Table 2. 

### 2.2. Three Frequently Used Open Datasets

Recording SSVEP signals takes effort, and many researchers choose to use open datasets to save time in obtaining EEG data and to train and validate their model. By using open datasets, it is easier to compare their methods with other methods because many other researchers have published their results based on the same dataset. Here, three frequently used open datasets by SSVEP deep learning research are summarized.

#### 2.2.1. Nakanishi Open Dataset

Nakanishi published their open dataset in 2015, making it the earliest and most frequently used SSVEP open dataset in deep learning research targeting SSVEP analysis [35]. In Nakanishi’s study, ten healthy subjects participated in the experiment. For each subject, the experiment consisted of 15 blocks, and in each block subjects were asked to gaze at one stimulus for 4 s and then complete 12 trials corresponding to all 12 targets. The stimuli flickered for 4 s on the monitor after a 1 s break for subjects to shift their gaze. The EEG data epochs were sampled at a sampling rate of 2048 Hz with eight electrodes, and later down-sampled to 256 Hz. All data were bandpass filtered from 6 Hz to 80 Hz with an infinite impulse response (IIR) filter. Considering a latency delay in the visual system, all data epochs were extracted with a 0.135 s delay after the stimulus onset. The Nakanishi 2015 open dataset can be obtained from https://github.com/NeuroTechX/moabb (accessed on 10 March 2023). 

**Table 2:** A detailed analysis of structures of 31 deep learning models used for SSVEP analysis. NM stands for not mentioned. C is the channel number of the dataset. T is the length of the segment data. GD stands for gradient descent. SGD stands for stochastic gradient descent. ReLU stands for rectified exponential linear unit. Other abbreviations are unique representations in the original paper, so please refer to the reference.

References	Year	Dataset	Preprocessing	Architecture	CNN Kernel Size	Pooling or Dropout	Training Method	Activation Function	Data Size Per Channel	Model Accuracy	Best Baseline Method and Accuracy
Cecotti [36]	2011	Author prepared	No	[128 × 6] × [6 × 128] × [6 × 112] × [6 × 12] × 100 × 5	1 × 6, 16 × 1	No	NM	Sigmoid	128 Hz × 6000 s = 768,000	0.9561	MEC (0.9035)
Bevilacqua et al. [37]	2014	Author prepared	No	[512 × 4] × [4 × 512] × [4 × 3] × 3	1 × 4	No	GD	Sigmoid	256 Hz × 576 s = 147,456	0.875 ± 0.076	SNR (0.695 ± 0.14)
Thomas et al. [38]	2017	Oikonomou et al. [39]	Welch method [40]	NM	1 × 4	Dropout	NM	ReLU	NM	0.6903	SVMG SFS (0.6609)
Kwak et al. [17]	2017	Author prepared	Filter, FFT	[120 × 8] × [8 × 120] × [8 × 110] × 5	1 × 8, 11 × 1	No	GD	Sigmoid	1000 Hz × 2500 s = 2,500,000(static)	99.28 ± 0.45	CCA-KNN (0.9770 ± 0.0285)
				[120 × 8] × [8@120] × [8 × 110] × 3 × 5	1 × 8, 11 × 1					97.83 ± 1.31	
				[960] × [500] × [100] × 5	No					98.44 ± 0.92	
Aznan et al. [41]	2018	Author prepared	Filter	[9] × [16 × (CNN)] × [600] × 4	NM	Max pooling and dropout	Adam [42]	ReLU	500 Hz × 480 s = 240,000	0.78 ± 0.1	SVM (0.51 ± 0.06)
Lawhern et al. [23]	2018	Gordon et al. [43]	No	[C × T] × [F1 × C × T] × [D × F1 × 1 × T] × [F2 × 1 × T] × N	1 × 64, C × 1, 1 × 16	Average pooling and dropout	Adam [42]	ELU	Not applied	Not applied	Not applied
Waytowich et al. [44]	2018	Nakanishi et al. [35]	No	[C × T] × [F1 × C × T] × [F2 × T//4] × [F2 × T//32] × N	1 × 256, 1 × 16	Average pooling and dropout	Adam [42]	ELU	256 Hz × 7200 s = 1,843,200	0.8	CCA(NM)
Nguyen [45]	2018	Author prepared	FFT	1D CNN layer × 1D CNN layer × 128 × 3 × 5	NM	Max pooling	Adam [42]	ReLU, Tanh	128 Hz × 1680 s = 215,040	0.9737 ± 2.86	CCA (0.91)
Kobayashi et al. [46]	2019	Author prepared	NM	1 × 50 (LSTM) × 70 (LSTM) × 5	No	Dropout	Adam [42]	NM	256 Hz × 400 s = 10,2400	0.968	k-NN (0.836)
Podmore [47]	2019	Wang et al. [48]	Normalization	[10 × 1500] × [736 × 100] × [354 × 100] × [163 × 100] × [34 × 100] × [2 × 100] × 40	10 × 30, 1 × 30	Max pooling	Adam [42]	ReLU	250 Hz × 9000 s = 2,250,000	0.8675	1DSCU (0.7473)
Ravi et al. [49]	2019	Author prepared	FFT	[C × 220] × [2C × 1 × 220] × [2C × 1 × 211] × N	1 × 10, 1 × 10	Dropout	SGD with Momentum	ReLU	1200 Hz × 4704 s = 5,644,800	0.7942	M-CNN (0.696)
		Nakanishi et al. [35]							256 Hz × 7200 s = 1,843,200	0.816	M-CNN (0.706)
Li et al. [50]	2020	Wang et al. [48]	Filter	{{[T × C] × [16 × T × C] × [T × C] × [T × 1]}||{[C × T × F1] × [40 × T × F1] × [T × F1]}} × N × N	9 × C, 1 × C, 1 × C, 9 × 1, 9 × 1	No	Adam [42]	No	250 Hz × 42,000 s = 10,500,000	0.9388(1 s)	TRCA (0.9299)
Ravi et al. [51]	2020	Author prepared	Filter, FFT	[C × F] × [2C × 1 × F] × [2C × 1 × (F-9)] × N	C × 1, 1 × 10	Dropout	SGD with Momentum	ReLU	1200 Hz × 7056 s = 8,467,200	0.816 ± 0.123	M-CNN (0.735 ± 0.161)
		Nakanishi et al. [35]							256 Hz × 7200 s = 1,843,200	0.816 ± 0.18	M-CNN (0.705 ± 0.22)
Dang et al. [52]	2021	Author prepared	FFT	[*p* × $l_{1}$] × [1 × $l_{1}$ × $N_{1}$] × [1 × $\left(l_{1}-m_{1}+1\right)\times N_{2}$] × $N_{3}$ × N	*p* × 1, 1 × $m_{1}$	Max pooling, Dropout	Adam [42]	ReLU	1000 Hz × 4800 s = 4,800,000	0.9723	CCA (0.9343)
Bassi et al. [53]	2021	Wang et al. [48]	FFT	[X × 64] × [X × 128] × [X × 256] × [X × 256] × [X × 512] × [X × 512] × 512 × 2	NM	Max pooling, Dropout	SGD with momentum	ReLU	250 Hz × 42,000 s = 10,500,000	0.822	DCNN (0.803)
Zhu et al. [54]	2021	Kwak et al. [55]	No	5 × EEGNet [21]		Average pooling and dropout	Adam [42]	ELU	250 Hz × 21,780 s = 5,445,000	0.8112(1 s)	CCA (0.5029)
Guney et al. [56]	2021	Wang et al. [48]	filter	[9 × 50 × 3] × [9 × 50 × 1] × [1 × 50 × 120] × [1 × 25 × 120] × [1 × 25 × 120] × 40	1 × 1,9 × 1,1 × 2,1 × 10	Dropout	Adam [42]	ReLU	250 Hz × 42,000 s = 10,500,000	0.84	NM
		Liu et al. [57]							250 Hz × 31,200 s = 7,800,000	0.7	NM
Ding et al. [58]	2021	Author prepared	Filter bank	[9 × 50] × [16 × 1 × 50] × [16 × 1 × 10] × [16 × 1 × 6] ×3 2 × 4	9 × 1, 1 × 50,1 × 5, 1 × 6	Dropout	Adam [42]	ELU	250 Hz × 3360 s = 840,000	0.8836 ± 0.0489	Compact-CNN (0.8298 ± 0.0622)
		Lee et al. [59]							250 Hz × 21,600 s = 5,400,000	0.7778 ± 0.0216	Compact-CNN (0.6779 ± 0.0234)
Pan et al. [60]	2022	Nakanishi et al. [35]	Filter bank	[C × T] × [2C × 1 × T] × [LSTM] × [8 × C × ((T-10)/2 + 1)/10] × [D1/5] ×N	1 × 10	Dropout	Adam [42]	PReLU	256 Hz × 7200 s = 1,843,200	0.8445 ± 0.1801	EEGNet (0.8078 ± 0.1838)
		Wang et al. [61]							250 Hz × 3200 s = 800,000	0.8422 ± 0.1586	C-CNN (0.8188 ± 0.137)
Li et al. [62]	2022	Author prepared	Wavelet Transform	[70 × 100 × 3] × × [100 × 70 × 30] × [100 × 70 × 50] × [50 × 35 × 20] × [50 × 35 × 50] × 5	15 × 10, 3 × 3, 3 × 3	Average pooling, Dropout	NM	ReLU	1000 Hz × 4800 s = 4,800,000	0.9675	Compact-CNN (0.95)
Li et al. [63]	2022	Author prepared	Filter, FFT	[3×78] × [16 × 3 × 78] × [32 × 1 × 78] × [32 × 1 × 78] × [64 × 1 × 76] × 4	3 × 1, 3 × 1, 1 × 3, 1 × 3	Dropout	Adam [42]	ReLU	NM	0.906	NM
Chen et al. [64]	2022	Nakanishi et al. [35]	Filter bank	[C × T] × 2 × {[2 × C × F] × [2 × C × F]} × [2FC] × N	2 × C, 2 × C	Dropout	SGD with momentum	GELU	256 Hz × 7200 s = 1,843,200	0.8837	CCNN (0.83)
		Wang et al. [48]							250 Hz × 42,000 s = 10,500,000	0.8319	CCNN (0.75)
Zhao et al. [65]	2022	Author prepared	No	[C × T] × [256 × 1 × $h_{1}$] × [128 × 1 × $h_{2}$] × [64 × 1 × $h_{3}$] × [8 × 1 ×$h_{4}$] × [8 × $h_{4}$] × 9	1 × 16, 1 × 32, 1 × 16, 1 × 2	Pooling and dropout	GD	ReLU	1000 Hz × 60,750 s = 60,750,000	0.8145	FBCCA (0.5867)
Avci et al. [66]	2022	Vilic [67]	Spectrogram	GoogLeNet	NM	NM	NM	NM	512 Hz×1440 s = 737,280	0.9128(in pairs)	NM
Bhuvaneshwari et al. [68]	2022	Author prepared	Filter	300 × [3 × 34] × [2 × 78] × [2 × 175] × [3 × 300] × [3 × 370] × 6	2 × 2, 3 × 3, 4 × 4, 5 × 5	Pooling, Dropout	Adam [42]	NM	NM	0.8891	NM
Israsena et al.[69]	2022	Wang et al. [48]	FFT	NM	5 × 5, 3 × 3	Max pooling, Dropout	Adam [42]	ReLU	250 Hz × 42,000 s = 10,500,000	0.7903	NM
Macias et al. [70]	2022	Author prepared	Filter, FFT	[30 × 3 0] × [10 × 21 × 21] × [80 × 7 × 7] × 490 × 4	9 × 9, 9 × 9	No	Adam [42]	ReLU	3,000 × 456 s = 1,368,000	0.9606	NM
Zhang et al. [71]	2022	Wang et al. [48]	Filter	{{LSTM × LSTM}||{LSTM × LSTM}} × correlation analysis×convolution×N	5 × 1	No	Adam [42]	Sigmoid, Tanh	250 Hz × 42,000 s = 10,500,000	0.9407 ± 0.1205	Conv-CA (0.9336 ± 0.1052)
		Zhu et al. [72]							250 hz × 24,480 s = 6,120,000	0.8796 ± 0.1082	eTRCA (0.8642 ± 0.1834)
Yao et al. [73]	2022	Nakanishi et al. [35]	Filter bank	3 × EEGNet [21]	1 × 256, 8 × 1	Average pooling and dropout	Adam [42]	Sigmoid	256 Hz × 7200 s = 1,843,200	0.82	EEGNet (0.8)
		Author prepared			1 × 256, 16 × 1	Average pooling and dropout	Adam [42]	Sigmoid	250 Hz × 40,500 s = 10,125,000	0.91	EEGNet (0.9)
Xiao et al. [74]	2022	Nakanishi et al. [35]	filter	{{[C × T] × [3 × C × T] × [3 × 1 × T] × [3 × T_1_] × [3 × T_1_]}||{[2N*_h_* × T] × [3 × 2N*_h_* × T] × [3 × 1 × T] × [3 × 1 × T]}} × 9 × 3	1 × 9, C × 1, 1 × 2, 1 × 1 × 9, 1 × 9, 2N*_h_* × 1, 1 × 2, 1 × 9	Dropout	Adam [42]	Tanh	256 Hz × 7200 s = 1,843,200	0.87(0.5 s)	FBeTRCA (0.928)
		Wang et al. [48]							250 Hz × 42,000 s = 10,500,000	0.81(0.5 s)	FBTDCA (0.841)
		Liu et al. [57]							250 Hz × 31,200 s = 7,800,000	0.694(0.5 s)	FBTDCA (0.671)
Paula et al. [75]	2023	Author prepared	CCA, recurrence plot	Dense161	NM	NM	SGD with momentum	NM	256 Hz × 4686 s = 1,199,616	0.97	Resnet101(0.95)

#### 2.2.2. Wang Open Dataset

Wang presented an open dataset which included a large number of subjects (8 experienced and 27 naïve, 35 in total) in 2017 [48]. For each subject, the experiment included 6 blocks, each containing 40 trails corresponding to 40 stimuli. The visual stimuli flickered for 5 s after a 0.5 s target cue, and there was a 0.5 s rest time before the next trial began. The EEG data epochs were recorded at a sampling rate of 1000 Hz with 64 electrodes and later down-sampled to 250 Hz. The Wang 2017 open dataset can be obtained from http://bci.med.tsinghua.edu.cn/download.html (accessed on 10 March 2023).

#### 2.2.3. BETA Open Dataset

Liu presented the BETA open dataset including 70 subjects performing a 40-target cued-spelling task in 2020 [57]. The 70 subjects all participated in the second round of the Brain–Computer Interface 2018 Olympics in China, and none of them were naive to the SSVEP-BCI. The experiment included 4 blocks each containing 40 trials corresponding to 40 stimuli. The visual stimuli flickered for 2 s for the first 15 participants and 3 s for the remaining 55 participants. There was a 0.5 s cue time before the flickering and a 0.5 s rest time after the flickering. The EEG data epochs were recorded at a sampling rate of 1000 Hz and later down-sampled to 250 Hz. A bandpass filtering between 3 and 100 Hz was conducted to remove the environmental noise. The BETA 2020 open dataset can be obtained from http://bci.med.tsinghua.edu.cn/download.html.

### 2.3. Data Preprocessing

Data preprocessing can enhance the performance of the model by making the features easier to extract. Common techniques including frequency filters, time-frequency transforms, and filter banks are often implemented in SSVEP analysis using deep learning.

#### 2.3.1. Frequency Filters

By applying frequency filters, noise can be removed from the data. Many open datasets consist of already filtered data using frequency filters including bandpass filters and notch filters. In Nakanishi’s open dataset, a bandpass filter from 6 Hz to 80 Hz was applied to remove low-frequency noise and high-frequency noise, as the stimulus frequencies between 9.25 Hz and 15.25 Hz together with their harmonics were included. In Wang’s open dataset, a notch filter at 50 Hz was applied to remove the power-line noise in the recording. In the BETA open dataset, a bandpass filter from 0.15 Hz to 200 Hz and a notch filter at 50 Hz were applied. Many researchers apply frequency filters in their own datasets as well, as shown in Table 2.

#### 2.3.2. Time-Frequency Transform

The implementation of a time-frequency transform can make the frequency features easier to extract by the deep learning models. When time domain signals are used as the input, a more complex model is usually required to extract features, while the neural networks with frequency domain input data have a relatively simpler structure. In SSVEP deep learning research, Fast Fourier Transform (FFT) is the most widely used time-frequency transform. Kwak implemented FFT to the input data and transformed input time domain data into 120 frequency samples through 8 channels [17]. Nguyen applied FFT to single-channel data to reduce the computation time of the system and use it as the only input into a 1D CNN model for SSVEP classification [45]. Ravi applied FFT to transform 1200 time-domain samples into 110 frequency components per data segment [49]. In these studies, FFT also caused the input to contain fewer data points, thus reducing the impact of overfitting, as training data were limited.

In some studies, FFT data were processed before feeding into the model to enhance model performance. In 2020, Ravi found that CNN models using complex spectrum features that were concatenated by the real part and the imaginary part of the complex FFT have higher accuracies than the same models using the magnitude spectrum of FFT as the input [51]. Dang took FFT as the input and intercepted the spectrum sequences of the fundamental waves and two harmonics and used them as parallel inputs into the CNN model to enhance the model’s performance [27].

#### 2.3.3. Filter Bank

Filter bank analysis performs sub-band decompositions with multiple filters that have different pass-bands. In 2015, Chen proposed a filter bank canonical correlation analysis (FBCCA) that incorporates fundamental and harmonic frequency components together to enhance the detection of SSVEP. By adding a filter bank to CCA analysis, FBCCA significantly outperformed CCA [28], which proved the filter bank to be an efficient data preprocessing method. Recently, researchers found that filter bank analysis can be implemented to process the inputs of deep learning models as well.

Ding built and compared two CNN models in 2021, one with a filter bank and one without a filter bank. Ding found that by adding a filter bank analysis to the input of the CNN model, the classification accuracy displayed a 5.53% increase in his own dataset on average, and a 5.95% increase in a public dataset [58]. In 2022, Pan leveraged four filter banks ranging from 8×m to 80 Hz for the input data before inserting them into a CNN-LSTM network, where m ∈ {1,2,3,4} [60]. Chen also implemented three filter banks to enhance a transformer-based model’s performance, which was named FB-SSVEPformer. Chen also found that, compared to using two or four filter banks, using three filter banks provided the best performance [64]. Yao built three filter banks and then fed the input to three EEGNets used as sub-networks separately before merging the features together [73]. These studies showed that a filter bank is an effective tool to process the SSVEP input and make frequency features easier to extract by the deep learning models.

## 3. Model Structure

Frequently used deep learning models can be generally categorized into three categories: fully connected neural networks, convolutional neural networks (CNNs), and recurrent neural networks (RNNs) [76]. CNNs have convolution layers in the network, meaning they have fewer connections than fully connected neural networks and generally need less computation power than fully connected neural networks [77]. CNNs are generally less prone to overfitting than fully connected neural networks when training data are limited. RNNs are different from fully connected neural networks and CNNs, as RNNs have memory of the input. RNNs perform the same function for every input data while the output of the current input depends on the previous computation. Long short-term memory (LSTM) is one kind of RNN and has been used in SSVEP signal classification.

### 3.1. Aritificial Neural Networks (ANNs)

ANNs are also known as feed forward neural networks, as they only have a forward direction that information will flow through the network with no turning back. ANNs have the advantage of generalized classification or predicting ability; with a properly designed structure, ANNs’ performance enhances with subsequent training data. The disadvantage of ANNs is that it takes copious amounts of data to train an ANN, and this may not be viable in many data-insufficient areas such as BCIs. Additionally, there is no specific rule in the structure design of ANNs, which makes the design process more like trial and error, and also time consuming.

Of the 31 studies that used deep learning models to analyze SSVEP signals, only one study used an ANN model. In 2016, Kwak built three models to classify SSVEP signals, a CNN-1 with two convolutional hidden layers, a CNN-2 with two convolutional hidden layers and one fully connected hidden layer, and a fully connected neural network with two fully connected hidden layers. Kwak found that CNN-1 outperformed the other two, and the DNN model’s performance was the worst of the three. Kwak deduced that the reason that CNN-1 outperformed all other methods was because of its low complexity with a simple structure, which was effective in his training-data-insufficient condition [17].

### 3.2. Recurrent Neural Networks (RNN)

RNN is known for its capability of storing temporary memory in the network, it has advantages in processing sequential information, especially in language translation, speech recognition, etc. The disadvantage of RNN is it is generally hard to train, both in time and in complexity.

LSTM is a type of RNN that has higher memory power and is thus able to learn long-term dependencies. Kobayashi first applied LSTM in 2019 to decode SSVEP signals in controlling drones and achieved an accuracy of 96.8%, which was significantly better than using FFT combined with machine learning methods such as decision tree (DT), support vector machine (SVM), linear discriminant analysis (LDA), and k-nearest neighbor nonparametric regression (k-NN) [46]. In 2022, Pan merged LSTM and a CNN together in his LSTM-CNN model and achieved the highest classification accuracies in two datasets. In Pan’s LSTM-CNN model, a BiLSTM module was added after a one-dimensional convolution module used for temporal filtering [60]. Zhang proposed a bidirectional Siamese correlation analysis (bi-SiamCA) model that used two LSTM layers to extract features of the EEG signal and reference signal and then analyzed their correlation before feeding to a convolution layer [71].

### 3.3. Convolutional Neural Networks (CNNs)

CNNs are deep learning models that use convolutional layers and, in most cases, use pooling layers as well. The convolutional layers can extract features through convolutional kernels, and pooling layers can increase the observation field of the hidden layers. The advantage of CNNs is that they have weight sharing mechanisms, and thus the computation cost of CNNs is low compared to other deep learning models, and they can detect features without human intervention. The disadvantage of CNNs is that they usually require large amounts of data to train, and the complex structure of a CNN requires high computational power to train.

Nearly all of the studies that are included in this survey used convolutional layers in their model. This showed the effectiveness of convolutional layers in SSVEP analysis. One possible explanation of convolutional layers’ popularity could be that convolutional layers take advantage of the local spatial coherence of SSVEP signals in the time domain or frequency domain, allowing the model to have lower weight and to be more easily trained in an SSVEP dataset. The structural design of CNN models can be seen in Table 2.

#### 3.3.1. Number of Convolutional Layers

Some studies suggest that CNN models with more convolutional layers have better performance. Aznan found that although the shallow model with one convolutional layer worked well for subject S01, with an accuracy of 96 ± 2%, when the model was applied to subject S04, whose EEG data were absent from the training dataset, the classification accuracy dropped to 59%. However, by changing the convolutional layer number from one to five, the classification accuracy of subject S04 increased to 69%, which suggested that perhaps a deeper model is required to perform the inter-subject SSVEP classification [41]. Podmore built a CNN model with five hidden layers and achieved 86% offline accuracy of classification. In his experiment, his model was better than FBCCA when using only data from three channels, but worse than FBCCA when more channels were used. Additionally, Podmore demonstrated that his model had better performance than 1DSCU, which is a CNN model with only one hidden layer [47]. Zhao applied a CNN model with five hidden layers on the classification of AR-SSVEP and found it to be significantly more accurate than ensemble-TRCA, CCA, and FBCCA [65].

Some studies suggest that CNN models with fewer convolutional layers have better performance. Kwak implemented two kinds of CNN and one DNN neural network on the decoding of SSVEP signals. Kwak found that CNN-1, a convolutional neural network with two hidden layers, outperformed CNN-2, which had three hidden layers, and DNN, suggesting that more CNN layers may not be good for the model [17]. Based on these studies, it is observed that the models’ performance is influenced by the number of convolutional layers, but there is not a linear relationship between the number of convolutional layers and the performance of the model.

#### 3.3.2. Size of CNN Kernels

The kernels are used to convolve on the previous layer’s output. A smaller kernel tends to collect more local information, while a larger kernel tends to collect more global information. In SSVEP analysis, most of the models use one-dimensional convolution, and thus the kernel size is 1×N. Here, the kernel sizes of CNN models are summarized in Figure 2B, and the details of the kernel sizes are shown in Table 2. From Figure 2B, it can be observed that small one-dimensional kernels of sizes below 1 × 25 are preferred in these studies.

### 3.4. Pooling Layer and Dropout

**Figure 3:**
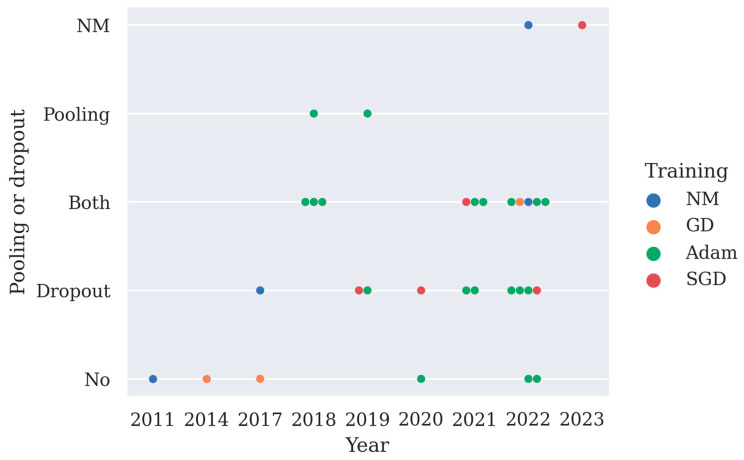
A summary of the implementation of pooling layers or dropout in 31 deep learning studies and their training methods, as shown in Table 2 (NM stands for not mentioned, GD stands for gradient descent, SGD stands for stochastic gradient descent with momentum, pooling is short for pooling layer).

Both a pooling layer and dropout can reduce the computation cost of the model and overfitting. Max pooling layer or average pooling layer are often used after the convolution layer, and they help to reduce the spatial size of the convolved features as well as overfitting by providing an abstracted representation of the features. Dropout works by randomly zeroing some of the connections in the network, thus overfitting and some of the computation costs are reduced. Deep learning models often adopt pooling layers and dropout, especially in CNN models and LSTM models. Here, the implementation of pooling layers and dropout in deep learning models is summarized in Figure 3. From Figure 3, it can be observed that since 2021 more studies have chosen to use both pooling layers and the dropout technique to minimize overfitting in their models.

### 3.5. Training Method

Gradient descent (GD) is the earliest method in deep learning to minimize the loss function of the model and optimize the model’s weights. The disadvantage of GD is that it can easily be trapped at local minimal weights or saddle points instead of global minimal weights and can stop optimizing the model.

Stochastic gradient descent with momentum (SGD) implements the exponential moving average (EMA) to accumulate previous weight changes and have a better chance of escaping local minimal and saddle points. SGD with momentum has the disadvantage of not being able to adjust the step size to approach local minimal points in greater depth instead of oscillating between slopes. To solve this problem, root mean square propagation (RMSProp) adjusts the step size to avoid bouncing between ridges and move towards the minima.

Adam, which is short for adaptive moment optimization, combines the heuristics of both RMSProp and momentum. It is considered to be the optimum training method of deep learning models. From Figure 3, it can be observed that Adam is the most frequently used training method in deep learning research.

### 3.6. Activation Function

An activation function is used in the neuron of the deep learning model to add non-linearity to the model and allows the model to abstract non-linear features from the input data. There are various types of activation functions, and they all have their advantages and disadvantages.

A sigmoid function mimics the probability value and gives a normalized output, which is easy to understand and is often used in shallow networks. The disadvantage of a sigmoid function is that it can cause the vanishing gradient problem, and its exponential calculation is slow for computers. Tanh provides stronger gradients than rectified linear unit (ReLU), and it has a zero-centered output, which facilitates back-propagation. The disadvantage of Tanh is that it also has the vanishing gradient problem just like the sigmoid function. ReLU makes the computation easier, and it can significantly improve the training speed of the deep learning model. ReLU also does not have the vanishing gradient problem. The disadvantage of ReLU is that when the input is negative, ReLU is inactive, thus it can generate dead neurons. The Gaussian error linear unit (GELU) was invented in 2016 and is very similar to ReLU; however, it was validated to provide improvement across computer vision, natural language processing, and speech tasks compared to ReLU [78]. The disadvantage of GeLU is that it has a complex computation. Parametric rectified linear unit (PReLU) is an improved version of ReLU, it has a small slope for negative values, and thus prevents the dying ReLU problem in which the ReLU neuron is stuck at the negative side and keeps outputting zero. PReLU is one of the most advanced activation functions in deep learning and appeared only once in our survey, in a paper published in 2022. The softmax function is used in almost every multivariate classification deep learning model as the output layer, as it turns the output vector into a vector that contains only positive numbers between 0 and 1, and it has an output sum of 1. This means that the output can be interpreted as probabilities.

**Figure 4:**
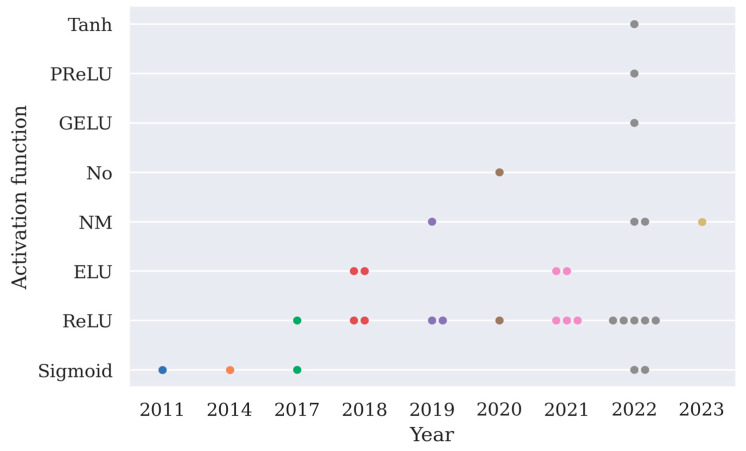
A summary of different types of activation functions used in 31 deep learning models as shown in Table 2 (PReLU stands for parametric rectified linear unit, GELU stands for Gaussian error linear unit, ELU stands for exponential linear unit, No means an activation function is not used in the model).

The choice of activation function is arbitrary except for the output layer, and here the implementation of activation functions except for the softmax function is summarized in Figure 4. From Figure 4, it can be observed that ReLU is the most frequently implemented activation function.

## 4. Discussion

As Table 2 shows, deep learning models are increasingly being employed to classify SSVEP signals with the progressive advancement of deep learning techniques. For a deep learning method that can successfully classify an SSVEP signal, the process of design and the performance measures are crucial.

### 4.1. Design of Model

The design of deep learning models in classifying SSVEP signals involves gaining SSVEP datasets, designing models, and enhancing model performance. In Section 2, the data length of 31 SSVEP deep learning studies is analyzed, and three commonly used open datasets are provided. This provides information for the researchers who are unsure of the data length that they should use to test their deep learning model.

Some researchers choose small datasets for their training and then use data augmentation to expand the dataset. Kobayashi’s dataset contained only 400 s of SSVEP signal data. To expand the training data, Kobayashi split the 20 s of data into 923 segments with a 0.0195 s shift and a 2 s length. This expanded the 20 s of data into 1846 s of data for the model and allowed the model to be well trained [46]. Other data augmentation techniques such as SpecAugment have also been used to augment EEG data. SpecAugment was used initially in speech recognition and turned out to be effective in expanding SSVEP data [53].

Choosing an open dataset rather than self-collected data may be a better choice because many researchers have already published their models’ performance based on open datasets, making it convenient to compare results. Additionally, this saves a lot of time in collecting data. Many researchers choose to use self-collected data together with public datasets or even multiple open datasets to validate their models’ performance objectively, as shown in Table 2.

For the structural design of the deep learning model, CNN models are currently the most widely used models, and they will most likely perform well in future studies. CNN models’ weight sharing feature and minimization of computation make them easier to be trained and efficient at extracting spatial features of data, especially when FFT is performed on the input data. However, the design of CNN models includes choosing many hyperparameters, such as the number of convolutional layers, the size of kernels, the activation function, the implementation of pooling layers or dropout, etc.

In this survey, detailed structures of 26 uniquely designed CNN models are shown in Table 2, which provides information for researchers who want to design their own CNN models. Additionally, a general structure of a CNN model can be observed: an input layer consisting of channels × time points or FFT data points; two to three convolutional layers with pooling layers or dropout; a fully connected layer between the last convolutional layer and output layer; and an output layer which contains the same number of neurons as the number of stimuli.

In the choice of an activation function, a popular choice would be to use the ReLU function as the activation function in hidden layers and the softmax function as the output function. However, with the advent of GeLU and PReLU which prove to be better substitutes of ReLU the choice of GeLU and PReLU should be considered as a promising alternative.

When tuning hyperparameters for the model, optimizing algorithms can also be used. Bhuvanesshwari proposed an automated hyperparameter optimization technique using the Red Fox Optimization Algorithm (RFO) and compared its result with the results of four other optimization algorithms and found that the hyperparameters of a five-layer CNN optimized by RFO has the highest classification accuracy of 88.91% [68].

For the training of the deep learning models, Adam combines the advantages of RMSprop and momentum and is generally the best choice. This can be observed in the mass usage of Adam since 2018, as shown in Table 2.

Other than designing CNN models from scratch, some researchers choose to modify existing CNN models used in computer vision to classify the SSVEP signal. Avci converted an SSVEP signal into a spectrogram and routed it to GoogLeNet deep learning model for binary classification [66]. Paula encoded EEG data to images using time-series imaging techniques and then used four 2D-kernel-based CNNs in the computer vision field, including ResNet, GoogLeNet, DenseNet and AlexNet, to classify the SSVEP signal [75].

EEGNet, a compact convolutional neural network initially designed for classifying multiple BCI paradigms including P300 visual-evoked potential, error-related negativity responses (ERN), movement-related cortical potential (MRCP), and sensor motor rhythms (SMRs), has also been widely utilized as a basic module in CNN models. In Yao’s research, three EEGNets were used as sub-networks in his CNN model [73]. Likewise, Li modified EEGNet and applied transfer learning to initially train the model parameters [63]. Zhu applied an ensemble learning strategy to combine multiple EEGNet models with different kernel numbers together to enhance the classification accuracy of ear-EEG signals from 50.61% to 81.12% at a 1 s window length of the EEG signal. Zhu also demonstrated that the classification accuracy of the average ensemble model surpasses the accuracy of a single EEGNet model with different kernel numbers [54]. These studies show that EEGNet is an effective building block in CNN model design.

Schirrmeister showed that convolutional neural network design choices substantially affect decoding accuracies, especially that the implementation of batch normalization and dropout significantly increase accuracies, which also shows that recent advances in deep learning methods improve the performance of the model [22]. Thus, adding newly developed deep learning technology may be an effective way of enhancing model performance.

### 4.2. Performance Measure of the Model

Most of the studies chose accuracy as the measure of their model’s performance. The formula for calculating accuracy is shown below:

(1) $$P=\frac{N\text{\_}correct}{N\text{\_}total}$$

where P is the prediction accuracy, *N_correct* is the total number of correct predictions in the experiment, and *N_total* is the total number of predictions in the experiment.

The comparison between the newly developed model’s accuracy with existing methods’ accuracies based on the same dataset is valid, but the comparison of accuracy values across different studies based on different datasets is not valid. This is because the classification accuracy also depends on the number of stimuli, as more stimuli means lower probability in choosing the right stimulus by chance.

The accuracy also depends on whether the detection is inter-subject or intra-subject. Intra-subject detection is also known as user-dependent (UD) detection. In this case, the model is trained using the data of one single participant and validated on the same participant. Inter-subject detection is also known as user-independent (UI) detection, in which the model is trained using the data of multiple participants and validated on the novel unseen user’s data. Ravi demonstrated that UD-based training methods consistently outperformed UI methods when all other conditions were the same [51].

Another commonly used metric is information transfer rate (ITR), which measures the communication speed and quality of the BCI system. ITR is calculated by the following formula with units of bits/min, i.e.,

$$\mathrm{ITR}(P,T)=(log_{2}M+Plog_{2}P+\left(1-P\right)log_{2}[\frac{1-P}{M-1}])\frac{60}{T}$$

(2) $$=(log_{2}M)\;\frac{60}{T}\;(\mathrm{when}\;P=1)$$

where P is the prediction accuracy that lies between 0 and 1, M is the number of stimuli, and T is the stimulation duration in seconds. For the same model, P can be improved by using more sampled data points in each classification, thus making T longer, and causing the transmitting efficiency to fall. Shorter T will limit the data in each classification, causing P to fall. There is a trade-off in the optimization of ITR. In SSVEP deep learning research, few studies adopt ITR as a performance indicator. This could be due to the fact that deep learning models may need longer T for classification, and thus have low ITR compared to other traditional methods. In 2022, Guney designed a deep convolutional neural network that takes EEG from all the channels and achieved high ITRs of 265.23 bits/min and 196.59 bits/min with only 0.4 s of stimulation on two open datasets, which were the highest ITRs achieved on these two open datasets [56].

### 4.3. Limitations of This Survey

This survey aims at analyzing deep learning models used in SSVEP classification, and it does not cover deep learning models used for other brain signals such as P300, motor imagery (MI), etc. Although these brain signals are different to SSVEP, the deep learning models applied for these brain signals may be instructive to the designing of deep learning models for SSVEP classification. Additionally, recent advancements in other fields such as computer vision and language processing can aid the deep learning models used for SSVEP classification, which are not included in this survey.

## 5. Opening Challenges and Future Directions

Most of the studies on SSVEP signal classification are based on multi-channel data, as more data often means more potential features for the deep learning model. However, in real applications, wearing multi-channels EEG amplifiers is inconvenient and expensive. EEG devices using a lower number of electrodes ultimately translate to lower: (1) hardware costs, (2) hygiene risks, and (3) user discomfort [79]. Research on using one or a few channels of SSVEP data is meaningful. In 2022, Macias modified capsule neural networks (CapsNet) to classify SSVEP signals and achieved a classification accuracy of 98.02% on his own dataset using a single active channel [70].

Ear channels are also a good substitute for SSVEP signal detection rather than scalp channels in terms of convenience, unobtrusiveness, and mobility. In 2022, Israsena proposed a CNN structure with two convolutional layers to classify SSVEP signals from one scalp channel and two ear channels, T7 and T8. Israsena achieved 79.03% accuracy with a 5 s window from Oz and around 40% accuracy from T7 or T8 [69]. The accuracies were not high, and there is still room for improvement in detecting SSVEP signals from ear channels.

Most of the deep learning models use one-dimensional CNN kernels to extract spatial features of SSVEP signals in the time domain or in the frequency domain, which regards SSVEP signals as one-dimensional data recorded in multiple channels. This prevents the implementation of deep learning models in the computer vision area. However, in 2022, Avci demonstrated that by converting SSVEP signals into spectrograms, deep learning models in computer vision can be applied to SSVEP signal classification [66]. Avci’s work is inspirational and hopefully, in the future, by changing SSVEP signals into two-dimensional graph data, more models in the computer vision area can be implemented in SSVEP classification and demonstrated to be effective.

In 2015, the filter bank was proposed and used with CCA to improve the performance of CCA in classifying SSVEP. Multiple studies in this survey implemented filter banks to improve their deep learning models’ performance. There are other data preprocessing techniques that can be applied to deep learning models to enhance their performance as well. Future research can be conducted to study these techniques.

In summary, here are three future directions that researchers should pay attention to:

1. Using deep learning models to enhance the performance of SSVEP classification based on data from fewer or single channels, or ear channels to improve the SSVEP-based BCI’s practicality;
2. Implementing the latest deep learning models or techniques for the classification of SSVEP signals;
3. Trying different data preprocessing techniques to enhance deep learning models’ performance.

## 6. Conclusions

In this survey, 31 deep learning models in SSVEP-based BCI were examined in detail and analyzed. There are three key aspects to consider in the design of deep learning models, including the model input, model structure, and model performance measures. In the model input section, the data length is analyzed to provide a reference for the amount of data needed to train deep learning models in SSVEP classification. Then, three frequently used open datasets are presented. Frequently used data preprocessing methods using deep learning models including filters, FFT, and filter banks are also introduced. In the model structure section, different structures of deep learning models are analyzed as well as their basic components, such as activation function, kernel size, layer number, and training method. This provides information for the structural design of deep learning models. In the discussion section, the design and performance measures of deep learning models are discussed. In Section 5, current challenges and future directions are pointed out. More importantly, the design details of 31 deep learning models are summarized in Table 2 to offer a convenient and comprehensive reference for designing future deep learning models.

## Figures and Tables

**Table 1:** Reviews and surveys on using deep learning models to classify steady-state visual evoked potentials (SSVEPs). Here, EEG represents electroencephalography, fNIRS represents functional near-infrared spectroscopy, MEG represents magnetoencephalography, FE represents feature extraction, ML represents machine learning, and DL represents deep learning.

References	Year	Signal	Methods	DL Technique	DL Model Structures	DL Model Hyperparameters	DL Performance
Craik et al. [24]	2019	EEG	DL	Yes	Yes	Yes	Yes
Roy et al. [25]	2019	EEG	DL	Yes	No	No	Yes
Xu et al. [26]	2021	EEG	FE, ML, DL	Yes	No	No	Yes
Saeidi et al. [27]	2021	EEG	FE, ML, DL	Yes	No	No	Yes
Zhang et al. [28]	2021	EEG, fNIRS, MEG,	DL	Yes	No	No	Yes
Essa et al. [29]	2021	EEG, MEG, CT	FE, ML, DL	Yes	No	No	Yes
Zhang et al. [30]	2021	SSVEP	FE, ML, DL	Yes	No	No	No
Aggarwal et al. [31]	2022	EEG	FE, ML, DL	No	No	No	Yes
Pan et al. [32]	2023	SSVEP	DL	Yes	No	No	Yes
Hossian et al. [33]	2023	EEG	FE, ML, DL	Yes	No	No	Yes

## Data Availability

The data and code used for generating Figure 1, Figure 2A, Figure 2B, Figure 3, Figure 4 can be accessed at: https://github.com/DongcenXu/An-Analysis-of-Deep-Learning-Models-in-SSVEP-Based-BCI-A-Survey (accessed on 10 March 2023).

## References

[1] Wolpaw J.R., Birbaumer N., Heetderks W.J., McFarland D.J., Peckham P.H., Schalk G., Donchin E., Quatrano L.A., Rob-inson C.J., Vaughan T.M. (2000). Brain–computer interface technology: A review of the first international meeting. IEEE Trans. Rehabil. Eng..

[2] Donchin E., Spencer K., Wijesinghe R. (2000). The mental prosthesis: Assessing the speed of a P300-based brain–computer interface. IEEE Trans. Rehabil. Eng..

[3] Hwang H.J., Lim J.H., Jung Y.J., Choi H., Lee S.W., Im C.H. (2012). Development of an SSVEP-based BCI spelling system adopting a QWERTY-style LED keyboard. J. Neurosci. Methods.

[4] Lopes A.C., Pires G., Nunes U. (2013). Assisted navigation for a brain-actuated intelligent wheelchair. Robot. Auton. Syst..

[5] Carlson T., Millan J. (2013). Brain-controlled wheelchairs: A robotic architecture. IEEE Robot. Autom. Mag..

[6] Tangermann M., Krauledat M., Grzeska K., Sagebaum M., Blankertz B., Vidaurre C., Müller K.R. (2008). Playing pinball with non-invasive BCI. NIPS.

[7] Krepki R., Blankertz B., Curio G., Müller K.R. (2007). The Berlin Brain–computer Interface (BBCI)–towards a new communication channel for online control in gaming applications. Multimed. Tools Appl..

[8] Hochberg L.R., Serruya M.D., Friehs G.M., Mukand J.A., Saleh M., Caplan A.H., Branner A., Chen D., Penn R.D., Donoghue J.P. (2006). Neuronal ensemble control of prosthetic devices by a human with tetraplegia. Nature.

[9] Collinger J.L., Wodlinger B., Downey J.E., Wang W., Tyler-Kabara E.C., Weber D.J., McMorland A.J., Velliste M., Boninger M.L., Schwartz A.B. (2013). High-performance neuroprosthetic control by an individual with tetraplegia. Lancet.

[10] Abiri R., Borhani S., Sellers E.W., Jiang Y., Zhao X. (2019). A comprehensive review of EEG-based brain–computer interface paradigms. J. Neural Eng..

[11] Ibrahimi D., Mendiola-Santibañez J.D., Cruz Martinez E., Rodríguez-Reséndiz J., Pacheco I.T. (2021). Cortical activity at baseline and during light stimulation in patients with strabismus and amblyopia. IEEE Access.

[12] Sánchez-Reyes L.M., Rodríguez-Reséndiz J., Avecilla-Ramírez G.N., García-Gomar M.L., Robles-Ocampo J.B. (2021). Impact of eeg parameters detecting dementia diseases: A systematic review. IEEE Access.

[13] Farwell L.A., Donchin E. (1988). Talking off the top of your head: Toward a mental prosthesis utilizing event-related brain potentials. Electroencephalogr. Clin. Neurophysiol..

[14] Wolpaw J.R., McFarland D.J., Neat G.W., Forneris C.A. (1991). An EEG-based brain–computer interface for cursor control. Electroencephalogr. Clin. Neurophysiol..

[15] Cheng M. (2002). Design and implementation of a brain–computer interface with high transfer rates. IEEE Trans. Biomed. Eng..

[16] Wolpaw J.R. Brain–computer interfaces (BCIs) for communication and control. Proceedings of the 9th international ACM SIGACCESS Conference on Computers and Accessibility.

[17] Kwak N.-S., Müller K.-R., Lee S.-W. (2017). A convolutional neural network for steady state visual evoked potential classification under ambulatory environment. PLoS ONE.

[18] Başar E. (1988). EEG—Dynamics and evoked potentials in sensory and cognitive processing by the brain. Dynamics of Sensory and Cognitive Processing by the Brain.

[19] Wang Y., Wang R., Gao X., Hong B., Gao S. (2006). A practical VEP-based brain–computer interface. IEEE Trans. Neural Syst. Rehabil. Eng..

[20] Nicolas-Alonso L.F., Gomez-Gil J. (2012). Brain computer interfaces, a review. Sensors.

[21] LeCun Y., Bengio Y., Hinton G. (2015). Deep learning. Nature.

[22] Schirrmeister R.T., Springenberg J.T., Fiederer L.D.J., Glasstetter M., Eggensperger K., Tangermann M., Hutter F., Burgard W., Ball T. (2017). Deep learning with convolutional neural networks for EEG decoding and visualization. Hum. Brain Mapp..

[23] Lawhern V.J., Solon A.J., Waytowich N.R., Gordon S.M., Hung C.P., Lance B.J. (2018). EEGNet: A compact convolutional neural network for EEG-based brain–computer interfaces. J. Neural Eng..

[24] Craik A., He Y., Contreras-Vidal J.L. (2019). Deep learning for electroencephalogram (EEG) classification tasks: A review. J. Neural Eng..

[25] Roy Y., Banville H., Albuquerque I., Gramfort A., Falk T.H., Faubert J. (2019). Deep learning-based electroencephalography analysis: A systematic review. J. Neural Eng..

[26] Xu L., Xu M., Jung T.P., Ming D. (2021). Review of brain encoding and decoding mechanisms for EEG-based brain–computer interface. Cogn. Neurodyn..

[27] Saeidi M., Karwowski W., Farahani F.V., Fiok K., Taiar R., Hancock P.A., Al-Juaid A. (2021). Neural decoding of EEG signals with machine learning: A systematic review. Brain Sci..

[28] Zhang X., Yao L., Wang X., Monaghan J., Mcalpine D., Zhang Y. (2021). A survey on deep learning-based non-invasive brain signals: Recent advances and new frontiers. J. Neural Eng..

[29] Almabrok E., Kotte H. (2021). Brain Signals Analysis Based Deep Learning Methods: Recent advances in the study of non-invasive brain signals. arXiv.

[30] Zhang Y., Xie S.Q., Wang H., Zhang Z. (2020). Data analytics in steady-state visual evoked potential-based brain–computer interface: A review. IEEE Sens. J..

[31] Swati A., Chugh N. (2022). Review of machine learning techniques for EEG based brain computer interface. Arch. Comput. Methods Eng..

[32] Pan Y., Chen J., Zhang Y. (2023). A Survey of deep learning-based classification methods for steady-state visual evoked potentials. Brain-Appar. Commun. A J. Bacomics.

[33] Hossain K.M., Islam M., Hossain S., Nijholt A., Ahad M.A.R. (2022). Status of deep learning for EEG-based brain–computer interface applications. Front. Comput. Neurosci..

[34] Higgins J.P., Altman D.G., Gøtzsche P.C., Jüni P., Moher D., Oxman A.D., Savović J., Schulz K.F., Weeks L., Sterne J.A. (2011). The Cochrane Collaboration’s tool for assessing risk of bias in randomised trials. BMJ.

[35] Nakanishi M., Wang Y., Wang Y.T., Jung T.P. (2015). A comparison study of canonical correlation analysis based methods for detecting steady-state visual evoked potentials. PLoS ONE.

[36] Cecotti H. (2011). A time–frequency convolutional neural network for the offline classification of steady-state visual evoked potential responses. Pattern Recognit. Lett..

[37] Bevilacqua V., Tattoli G., Buongiorno D., Loconsole C., Leonardis D., Barsotti M., Frisoli A., Bergamasco M. A novel BCI-SSVEP based approach for control of walking in virtual environment using a convolutional neural network. Proceedings of the 2014 International Joint Conference on Neural Networks (IJCNN).

[38] Thomas J., Maszczyk T., Sinha N., Kluge T., Dauwels J. Deep learning-based classification for brain–computer interfaces. Proceedings of the 2017 IEEE International Conference on Systems, Man, and Cybernetics (SMC).

[39] Oikonomou V.P., Liaros G., Georgiadis K., Chatzilari E., Adam K., Nikolopoulos S., Kompatsiaris I. (2016). Comparative evaluation of state-of-the-art algorithms for SSVEP-based BCIs. arXiv.

[40] Oppenheim A.V., Buck J., Schafer R. (2001). Discrete-Time Signal Processing.

[41] Aznan N.K.N., Bonner S., Connolly J., Al Moubayed N., Breckon T. On the classification of SSVEP-based dry-EEG signals via convolutional neural networks. Proceedings of the 2018 IEEE International Conference on Systems, Man, and Cybernetics (SMC).

[42] Kingma D.P., Ba J. (2014). Adam: A method for stochastic optimization. arXiv.

[43] Gordon S.M., Lawhern V., Passaro A.D., McDowell K. (2015). Informed decomposition of electroencephalographic data. J. Neurosci. Methods.

[44] Waytowich N., Lawhern V.J., Garcia J.O., Cummings J., Faller J., Sajda P., Vettel J.M. (2018). Compact convolutional neural networks for classification of asynchronous steady-state visual evoked potentials. J. Neural Eng..

[45] Nguyen T.-H., Chung W.-Y. (2018). A single-channel SSVEP-based BCI speller using deep learning. IEEE Access.

[46] Kobayashi N., Ishizuka K. LSTM-based classification of multiflicker-ssvep in single channel dry-eeg for low-power/high-accuracy quadcopter-bmi system. Proceedings of the 2019 IEEE International Conference on Systems, Man and Cybernetics (SMC).

[47] Podmore J.J., Breckon T.P., Aznan N.K., Connolly J.D. (2019). On the relative contribution of deep convolutional neural networks for SSVEP-based bio-signal decoding in BCI speller applications. IEEE Trans. Neural Syst. Rehabil. Eng..

[48] Wang Y., Chen X., Gao X., Gao S. (2016). A benchmark dataset for SSVEP-based brain–computer interfaces. IEEE Trans. Neural Syst. Rehabil. Eng..

[49] Ravi A., Heydari N., Jiang N. User-independent SSVEP BCI using complex FFT features and CNN classification. Proceedings of the 2019 IEEE International Conference on Systems, Man and Cybernetics (SMC).

[50] Li Y., Xiang J., Kesavadas T. (2020). Convolutional correlation analysis for enhancing the performance of SSVEP-based brain–computer interface. IEEE Trans. Neural Syst. Rehabil. Eng..

[51] Ravi A., Beni N.H., Manuel J., Jiang N. (2020). Comparing user-dependent and user-independent training of CNN for SSVEP BCI. J. Neural Eng..

[52] Dang W., Li M., Lv D., Sun X., Gao Z. (2021). MHLCNN: Multi-harmonic linkage CNN model for SSVEP and SSMVEP signal classification. IEEE Trans. Circuits Syst. II Express Briefs.

[53] Bassi P.R., Rampazzo W., Attux R. (2021). Transfer learning and SpecAugment applied to SSVEP based BCI classification. Biomed. Signal Process. Control..

[54] Zhu Y., Li Y., Lu J., Li P. (2021). EEGNet with ensemble learning to improve the cross-session classification of SSVEP based BCI from Ear-EEG. IEEE Access.

[55] Kwak N.-S., Lee S.-W. (2019). Error correction regression framework for enhancing the decoding accuracies of ear-EEG brain–computer interfaces. IEEE Trans. Cybern..

[56] Guney O.B., Oblokulov M., Ozkan H. (2021). A deep neural network for ssvep-based brain–computer interfaces. IEEE Trans. Biomed. Eng..

[57] Liu B., Huang X., Wang Y., Chen X., Gao X. (2020). BETA: A large benchmark database toward SSVEP-BCI application. Front. Neurosci..

[58] Ding W., Shan J., Fang B., Wang C., Sun F., Li X. (2021). Filter bank convolutional neural network for short time-window steady-state visual evoked potential classification. IEEE Trans. Neural Syst. Rehabil. Eng..

[59] Lee M.H., Kwon O.Y., Kim Y.J., Kim H.K., Lee Y.E., Williamson J., Fazli S., Lee S.W. (2019). EEG dataset and OpenBMI toolbox for three BCI paradigms: An investigation into BCI illiteracy. GigaScience..

[60] Pan Y., Chen J., Zhang Y., Zhang Y. (2022). An efficient CNN-LSTM network with spectral normalization and label smoothing technologies for SSVEP frequency recognition. J. Neural Eng..

[61] Wang H., Zhang Y., Waytowich N.R., Krusienski D.J., Zhou G., Jin J., Wang X., Cichocki A. (2016). Discriminative feature extraction via multivariate linear regression for SSVEP-based BCI. IEEE Trans. Neural Syst. Rehabil. Eng..

[62] Wang H., Zhang Y., Waytowich N.R., Krusienski D.J., Zhou G., Jin J., Wang X., Cichocki A. (2022). DSCNN: Dilated Shuffle CNN model for SSVEP signal classification. IEEE Sens. J..

[63] Li P., Su J., Belkacem A.N., Cheng L., Chen C. (2022). Corrigendum: Multi-person feature fusion transfer learning-based convolutional neural network for SSVEP-based collaborative BCI. Front. Neurosci..

[64] Chen J., Zhang Y., Pan Y., Xu P., Guan C. (2022). A Transformer-based deep neural network model for SSVEP classification. arXiv.

[65] Zhao X., Du Y., Zhang R. (2022). A CNN-based multi-target fast classification method for AR-SSVEP. Comput. Biol. Med..

[66] Avci M.B., Sayilgan E. Effective SSVEP Frequency Pair Selection over the GoogLeNet Deep Convolutional Neural Network. Proceedings of the 2022 Medical Technologies Congress (TIPTEKNO).

[67] Signals V.A.A.s.-s.v.e.p.S. Dataset 2013. https://www.setzner.com/avi-ssvepdataset/.

[68] Bhuvaneshwari M., Kanaga E.G.M., George S. (2022). Classification of SSVEP-EEG signals using CNN and Red Fox Optimization for BCI applications. Proc. Inst. Mech. Eng. Part H J. Eng. Med..

[69] Israsena P., Pan-Ngum S. (2022). A CNN-Based Deep Learning Approach for SSVEP Detection Targeting Binaural Ear-EEG. Front. Comput. Neurosci..

[70] Macías-Macías J.M., Ramírez-Quintana J.A., Torres-García A.A., Chacón-Murguía M.I. Recognition of P300 Wave and SSVEP using a Capsule Neural Network. Proceedings of the 2022 19th International Conference on Electrical Engineering, Computing Science and Automatic Control (CCE).

[71] Zhang X., Qiu S., Zhang Y., Wang K., Wang Y., He H. (2022). Bidirectional Siamese correlation analysis method for enhancing the detection of SSVEPs. J. Neural Eng..

[72] Zhu F., Jiang L., Dong G., Gao X., Wang Y. (2021). An open dataset for wearable ssvep-based brain–computer interfaces. Sensors.

[73] Yao H., Liu K., Deng X., Tang X., Yu H. (2022). FB-EEGNet: A fusion neural network across multi-stimulus for SSVEP target detection. J. Neurosci. Methods.

[74] Xiao X., Xu L., Yue J., Pan B., Xu M., Ming D. (2022). Fixed template network and dynamic template network: Novel network designs for decoding steady-state visual evoked potentials. J. Neural Eng..

[75] De Paula P.O., da Silva Costa T.B., de Faissol Attux R.R., Fantinato D.G. (2023). Classification of image encoded SSVEP-based EEG signals using Convolutional Neural Networks. Expert Syst. Appl..

[76] Alfonso-Francia G., Pedraza-Ortega J.C., Badillo-Fernández M., Toledano-Ayala M., Aceves-Fernandez M.A., Rodri-guez-Resendiz J., Ko S.B., Tovar-Arriaga S. (2022). Performance Evaluation of Different Object Detection Models for the Segmentation of Optical Cups and Discs. Diagnostics.

[77] Ortiz-Echeverri C.J., Salazar-Colores S., Rodríguez-Reséndiz J., Gómez-Loenzo R.A. (2019). A new approach for motor imagery classification based on sorted blind source separation, continuous wavelet transform, and convolutional neural network. Sensors.

[78] Hendrycks D., Gimpel K. (2016). Gaussian error linear units (gelus). arXiv.

[79] Haselager P., Vlek R., Hill J., Nijboer F. (2009). A note on ethical aspects of BCI. Neural Netw..

